# TiO_2_-MXene/PEDOT:PSS Composite as a Novel Electrochemical Sensing Platform for Sensitive Detection of Baicalein

**DOI:** 10.3390/molecules28073262

**Published:** 2023-04-06

**Authors:** Shuya Xue, Min Shi, Jinye Wang, Jiapeng Li, Guanwei Peng, Jingkun Xu, Yansha Gao, Xuemin Duan, Limin Lu

**Affiliations:** 1 Flexible Electronics Innovation Institute (FEII), Jiangxi Science and Technology Normal University, Nanchang 330013, China; 2 Key Laboratory of Crop Physiology, Ecology and Genetic Breeding, Ministry of Education, Key Laboratory of Chemical Utilization of Plant Resources of Nanchang, College of Chemistry and Materials, Jiangxi Agricultural University, Nanchang 330045, China; 3 Shandong Liaocheng Ecological Environment Monitoring Center, Liaocheng 252000, China

**Keywords:** TiO_2_-MXene, oxygen vacancy, PEDOT:PSS, baicalein, electrochemical sensor

## Abstract

In this work, TiO_2_-MXene/poly (3,4-ethylenedioxythiophene): poly(styrenesulfonate) (PEDOT:PSS) composite was utilized as an electrode material for the sensitive electrochemical detection of baicalein. The in-situ growth of TiO_2_ nanoparticles on the surface of MXene nanosheets can effectively prevent their aggregation, thus presenting a significantly large specific surface area and abundant active sites. However, the partial oxidation of MXene after calcination could reduce its conductivity. To address this issue, herein, PEDOT:PSS films were introduced to disperse the TiO_2_-MXene materials. The uniform and dense films of PEDOT:PSS not only improved the conductivity and dispersion of TiO_2_-MXene but also enhanced its stability and electrocatalytic activity. With the advantages of a composite material, TiO_2_-MXene/PEDOT:PSS as an electrode material demonstrated excellent electrochemical sensing ability for baicalein determination, with a wide linear response ranging from 0.007 to 10.0 μM and a lower limit of detection of 2.33 nM. Furthermore, the prepared sensor displayed good repeatability, reproducibility, stability and selectivity, and presented satisfactory results for the determination of baicalein in human urine sample analysis.

## 1. Introduction

Baicalein (5,6,7-trihydroxyflavone), as a kind of natural flavonoid isolated from Scutellaria [1], has generally been utilized for the prevention and treatment of physical diseases [2,3]. According to previous reports, baicalein is widely used in anti-cancer, anti-inflammatory and anti-viral therapy [4,5,6]. Nevertheless, overdosing baicalein will inevitably result in serious side effects such as spasm, dizziness and coma [7]. Thus, the development of an accurate and sensitive method of detection for baicalein has aroused widespread attention. In this context, electrochemical sensors have attracted broad-scale research because of their high sensitivity, rapid response, simplified operation and low cost [8,9,10]. For an electrochemical sensing platform, selecting suitable electrode materials to promote detection performance it is a critical factor.

As a rapidly growing family of two-dimensional (2D) materials, MXene has gradually become a hot topic of important research in the field of electrochemical sensors [11]. Due to its metallic-like conductive nature, abundant surface groups and superior mechanical properties [12,13,14], MXene possesses excellent electrochemical properties and has been extensively applicated in electrochemical sensors [15]. Among various MXenes, Ti_3_C_2_ is the most widely used [16]. However, the strong self-stacking and agglomeration of the Ti_3_C_2_ nanosheets can sharply decrease the electrochemical performance [17], which will limit the practical application of the MXene. To overcome these drawbacks, the introduction of nanoparticles to modify the surface of MXene nanosheets is an effective and suitable approach to expanding the interlayer spacing of MXene nanosheets [18]. The edge Ti atom on the surface of Ti_3_C_2_T_x_ as a nucleation site could easy to generate TiO_2_ nanoparticles with rich oxygen vacancies (OVs) through the controlled oxidation of Ti_3_C_2_T_x_ [19]. The formed OV-TiO_2_ nanoparticles embedded in MXene sheets could effectively broaden the layer spacing. Moreover, the oxygen vacancies are conducive to promoting the formation of new active sites [20], which further boost the chemical adsorption capability. Despite such advantages, the partial oxidation of MXene inevitably decreases electrical conductivity. In addition, oxidation destroys the surface functional groups and reduces the water stability of MXene, which greatly restricts its applications in electrochemical sensing.

PEDOT:PSS is an extensively studied conductive polyelectrolyte complex with the structure of PEDOT-rich cores surrounded by a PSS-rich shell [21]. PEDOT:PSS has been used in the areas of thermoelectricity, organic photovoltaics, bioelectronics and sensing conductors [22,23,24,25] because of its high conductivity, appropriate stability and superior mechanical flexibility [26,27]. In the fabrication of electrochemical sensors, PEDOT:PSS could be used as conductive dispersant to enhance the dispersibility of other materials. Furthermore, the addition of nanomaterials makes it feasible to improve the physicochemical properties of PEDOT:PSS. As a proof of concept, the TiO_2_-MXene/PEDOT:PSS composite can be rationally designed. Until now, there has been no report on the fabrication of TiO_2_-MXene/PEDOT:PSS film as electrochemical sensor for baicalein detection.

In this work, we proposed a novel electrochemical sensing platform for baicalein determination based on a TiO_2_-MXene/PEDOT:PSS-modified glassy carbon electrode (GCE). The TiO_2_-MXene/PEDOT:PSS composite was prepared by oxygen vacancy-rich TiO_2_-MXene followed by direct ultrasonic mixing with PEDOT:PSS. VO-TiO_2_ embedded in MXene not only enhanced electron transfer but also possessed a relatively large surface area. The introduction of PEDOT:PSS film can further increase the stability and conductivity of the composite. Benefited by the synergistic effect between TiO_2_-MXene and PEDOT:PSS, the prepared sensor exhibited a wide linear working range and a low limit of detection (LOD) towards baicalein detection. Furthermore, the designed electrochemical sensor showed good selectivity and stability for baicalein detection, and it was also successfully applied to the detection of baicalein in real samples.

## 2. Results and Discussion

### 2.1. Characterization of Structure and Morphology

**Figure 1:**
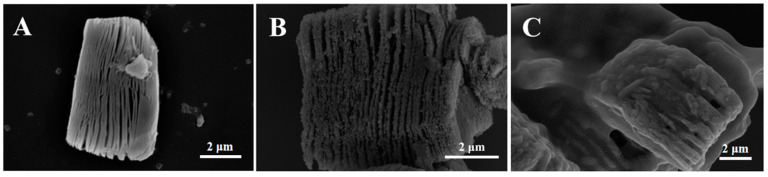
SEM images of (**A**) MXene, (**B**) TiO_2_-MXene and (**C**) TiO_2_-MXene/PEDOT:PSS.

The morphological characterizations of MXene, TiO_2_-MXene and TiO_2_-MXene/PEDOT:PSS were observed by scanning electron microscopy (SEM). As presented in Figure 1A, the pristine MXene possessed a typical layered accordion-like structure with a smooth surface. For TiO_2_-MXene (Figure 1B), the surface of MXene became rough, and there were many fine TiO_2_ nanoparticles on the surface or inside of the MXene nanosheets. The TiO_2_ nanoparticles could effectively hinder the stacking of MXene sheets and extend the interlayer distance, and thus more catalytic active sites were exposed. Simultaneously, the unique layered structure of MXene could be a large platform for loading TiO_2_ nanoparticles. After combining with PEDOT:PSS (Figure 1C), it could be seen that the surface of TiO_2_-MXene became smoother because of the great film-forming property of PEDOT:PSS. Additionally, the coating of PEDOT:PSS on the surface of TiO_2_-MXene did not change the morphology of TiO_2_-MXene.

**Figure 2:**
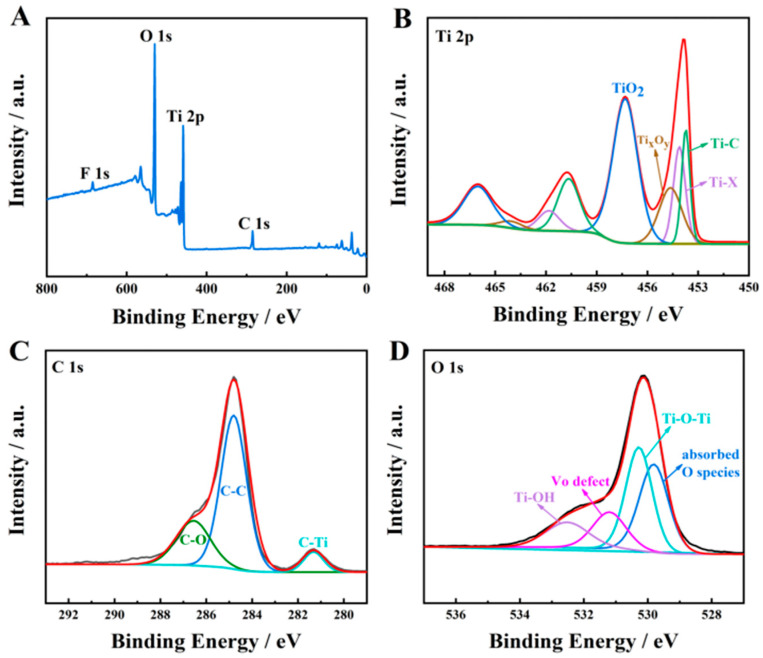
XPS survey spectrum of (**A**) TiO_2_-MXene, and high-resolution spectra of (**B**) Ti 2p, (**C**) C 1s and (**D**) O 1s.

The X-ray photoelectron spectroscopy (XPS) spectra were utilized to study the elemental composition on the surface of the TiO_2_-MXene composites. As displayed in Figure 2A, the XPS survey spectrum proved the presence of C, Ti, O and F elements in TiO_2_-MXene. In the Ti 2p spectrum (Figure 2B), the peaks at 453.7, 454.1, 454.6 and 457.3 eV were attributed to Ti-C, Ti-X from Ti (Ⅱ), Ti ions with reduced charge state (Ti_x_O_y_) and TiO_2_, respectively. The peak of TiO_2_ indicated that Ti_3_C_2_T_x_ partly transformed into TiO_2_ nanoparticles. Furthermore, the XPS spectrum of C1s (Figure 2C) exhibited three fitted peaks at 281.3, 284.8 and 286.6 eV, which could be assigned to C-Ti, C-C and C-O, respectively. According to the spectrum of O 1s (Figure 2D), the binding energies at 529.8, 530.3, 531.2 and 532.5 eV could be ascribed to absorbed O species, Ti-O-Ti, Vo defect and Ti-OH [28]. These results proved the successful preparation of oxygen vacancy-rich TiO_2_-MXene through annealing, which played a role in providing ample active sites and then enhanced the baicalein absorption.

### 2.2. Electrochemical Characterizations

**Figure 3:**
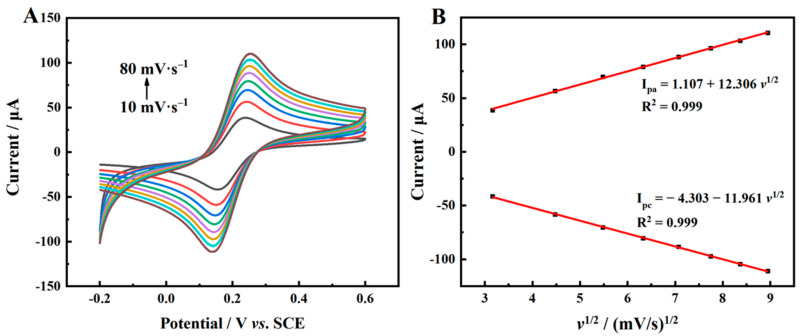
(**A**) CV responses of TiO_2_-MXene/PEDOT:PSS/GCE in 5 mM of [Fe(CN)_6_]^3−/4−^ solution containing 0.1 M of KCl at different scan rates; (**B**) The linear relationship between the peak current and *v*^1/2^.

The electrochemically active surface area (ECSA) was one of the key factors affecting the performance of electrode materials. The ECSA of the electrode material was estimated by cyclic voltammetry (CV) measurements with different scan rates (10–80 mV·s^−1^) in 5 mM [Fe(CN)_6_]^3−/4−^ containing 0.1 M KCl (Figure 3A). The value of the specific surface area was calculated by the Randles–Sevcik equation [29]:

(1) $$I_{p}=2.69\times 10^{5}\;n^{3/2}{\;\mathrm{ACD}}^{1/2}v^{1/2}$$

Herein, I_p_ represents the anodic or cathodic peak current, D (7.6 × 10^−6^ cm^2^·s^−1^) represents the diffusion coefficient, and *n* (*n* = 1) refers to the electron transfer number. A indicates the effective surface area of the working electrode, and C is the concentration of the probe molecule ([Fe(CN)_6_]^3−/4−^). According to the peak calibration equation displayed in Figure 3B, the ECSA of TiO_2_-MXene/PEDOT:PSS was calculated to be 0.1065 cm^2^. The obtained value was much higher than that of bare GCE (0.0707 cm^2^). The larger surface area could provide numerous active sites and reaction centers for the electrochemical redox of baicalein, thus improving the sensitivity towards baicalein detection.

**Figure 4:**
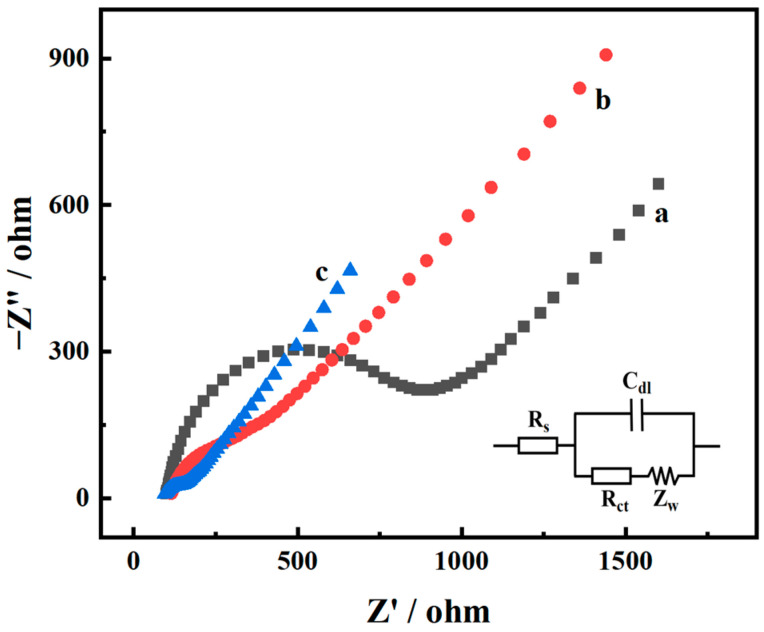
EIS of bare GCE (**a**), TiO_2_-MXene/GCE (**b**), and TiO_2_-MXene/PEDOT:PSS/GCE (**c**) in 5.0 mM [Fe(CN)_6_]^3−/4−^ solution containing 0.1 M KCl.

Electrochemical impedance spectroscopy (EIS) was used to investigate the interfacial properties of bare GCE, TiO_2_-MXene/GCE, and TiO_2_-MXene/PEDOT:PSS/GCE (Figure 4). The equivalent circuit used to simulate the Nyquist plots is presented in Figure 4, which consisted of the active electrolyte resistance (R_S_), the charge transfer resistance (R_ct_), the double-layer capacitance (C_dl_) and the Warburg impedance (Z_W_). In the Nyquist plots, the semicircle diameter represented the electron transfer resistance (R_ct_). On the basis of the EIS spectra of three different electrodes, the R_ct_ values of bare GCE (curve a), TiO_2_-MXene/GCE (curve b) and TiO_2_-MXene/PEDOT:PSS/GCE (curve c) were calculated to be 832.1 Ω, 294.2 Ω, and 73.9 Ω, respectively. The R_ct_ value of TiO_2_-MXene was much lower than bare GCE, indicating that the TiO_2_-MXene could improve the electrical conductivity of bare GCE. Moreover, the coating of PEDOT:PSS on TiO_2_-MXene made the R_ct_ value of TiO_2_-MXene/PEDOT:PSS/GCE further decrease. The lowest charge transfer resistance of TiO_2_-MXene/PEDOT:PSS/GCE suggested faster electron transfer kinetics for baicalein.

### 2.3. Electrochemical Behavior of Modified Electrodes

**Figure 5:**
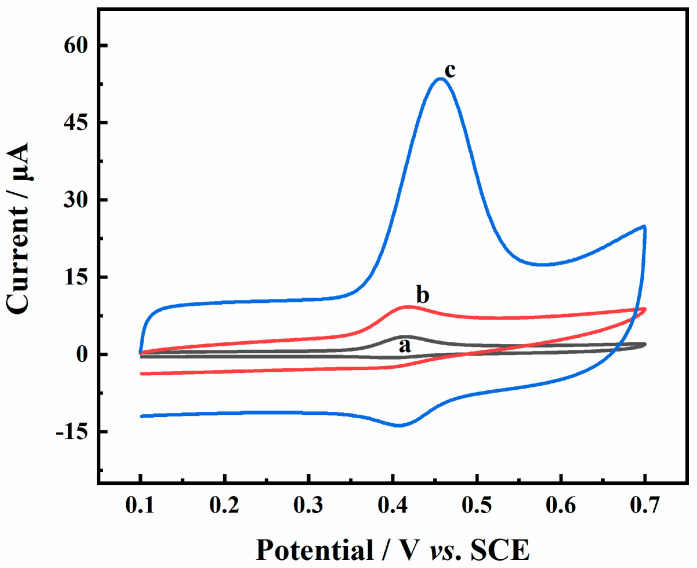
CVs of bare GCE (**a**), TiO_2_-MXene/GCE (**b**), and TiO_2_-MXene/PEDOT:PSS/GCE (**c**) in PB (0.1 M, pH 2.0) after accumulation in 10.0 μM baicalein for 180 s.

The electrochemical behaviors of baicalein at different modified electrodes were studied by CV measurement in 0.1 M phosphate buffer (PB, pH = 2.0) after accumulation in 10.0 μM baicalein for 180 s. As presented in Figure 5, there were very weak redox peaks for the unmodified GCE (curve a), which correspond to the electrochemical redox of baicalein. For TiO_2_-MXene/GCE (curve b), a high background current and larger redox current peaks can be observed, because TiO_2_-MXene with oxygen vacancies could effectively adsorb baicalein and the electrocatalysis of TiO_2_-MXene could favorably promote the redox of baicalein. Furthermore, TiO_2_-MXene/PEDOT:PSS/GCE (curve c) presented much larger redox currents. The excellent electrocatalytic performance mainly benefited from the synergy of TiO_2_-MXene and PEDOT:PSS, where oxygen vacancy-rich TiO_2_-MXene provided a large number of active sites and the surface of PEDOT:PSS further enhanced the electron transport ability and catalytic property of the composite. PEDOT:PSS films successfully established a conductive bridge between TiO_2_-MXene nanosheets, which exhibited promising electrochemical behavior for accelerating the oxidation of baicalein. Therefore, by combining highly conductive PEDOT:PSS with TiO_2_-MXene, the conductivity of the composite can be greatly improved, and the sensing signals of baicalein can be significantly amplified. These results revealed the remarkable properties of TiO_2_-MXene/PEDOT:PSS for baicalein detection.

### 2.4. Optimizations of Analytical Parameters

To ensure the optimal analytical performance of the sensor, different experimental parameters such as the accumulation time, pH of supporting electrolyte and volume of electrode modification material were investigated.

**Figure 6:**
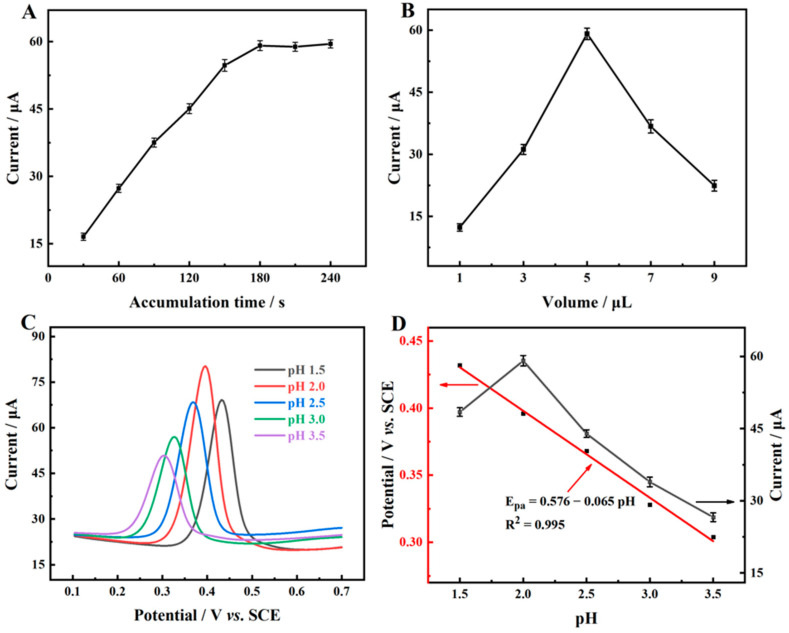
(**A**) Effect of accumulation time on current response; (**B**) Effect of the volume of TiO_2_-MXene/PEDOT:PSS on current response; (**C**) DPV of 10.0 μM baicalein determined by TiO_2_-MXene/PEDOT:PSS/GCE in PB (0.1 M) with different pH values; (**D**) Effect of pH value on the I_pa_ and E_pa_.

The influence of accumulation time on the signal response of baicalein was investigated by differential pulse voltammetry (DPV). From Figure 6A, it could be found that the oxidation peak currents gradually increased when the accumulation time raised from 30 s to 180 s. However, the peak current remained stable when the time continued to increase, suggesting that the adsorption of baicalein at the TiO_2_-MXene/PEDOT:PSS electrode tended to be saturated. However, the longer accumulation time resulted in the prolonging of the operational time. Therefore, 180 s was selected as the most appropriate accumulation time for the following detection.

Furthermore, the volume of modified materials also affects the performance of the electrochemical sensor. The effect of the volume of the TiO_2_-MXene/PEDOT:PSS composite was investigated by DPV (Figure 6B). The peak current of baicalein gradually increased as the volume of TiO_2_-MXene/PEDOT:PSS increased from 1 µL to 5 µL. Nevertheless, when we further increased the modifier volume, the peak current decreased, indicating that a much thicker TiO_2_-MXene/PEDOT:PSS composite hindered the electron transfer and inhibited the electrocatalytic activity. Thus, 5 µL was chosen as the optimum material volume for further experiments.

The effect of electrolyte pH value on the DPV response of TiO_2_-MXene/PEDOT:PSS/GCE with 10 μM baicalein was examined, as the pH of PB (0.1 M) ranged from 1.5 to 3.5. As depicted in Figure 6C, the oxidation peak potential (E_pa_) shifted negatively with the increase in pH values, which implied that protons were involved in the redox reactions. E_pa_ versus pH value presented a good linearity, with a linear regression equation of E_pa_ = 0.576 − 0.065 pH (R^2^ = 0.995) (Figure 6D). The obtained slope value (−65 mV·pH^−1^) was in proximity to the theoretical value (−59 mV·pH^−1^), suggesting that the same number of electrons as protons were participating in the electrochemical reaction of baicalein. Moreover, among all of the pH ranges investigated herein, the peak current of baicalein reached the maximum with PB at pH 2.0. Hence, pH 2.0 was adopted as the optimal pH value of PB for the subsequent electrochemical detection.

### 2.5. Kinetics Studies

**Figure 7:**
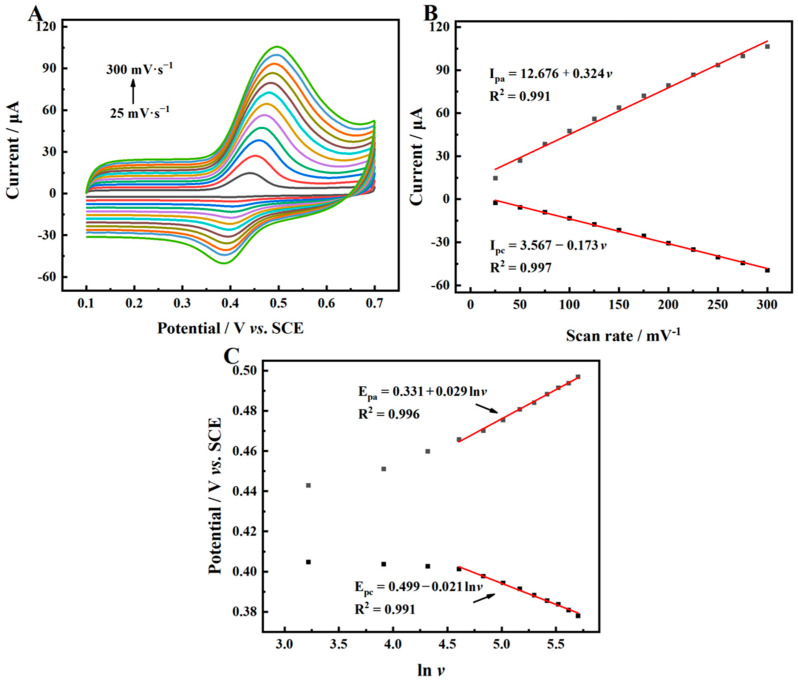
(**A**) CVs of 10.0 μM baicalein on TiO_2_-MXene/PEDOT:PSS/GCE in 0.1 M PB (pH 2.0) at different scan rates (*v*) from 25 to 300 mV·s^−1^; (**B**) The relationship between I_p_ and *v*; (**C**) The relationship between E_p_ and ln*v*.

To investigate the redox mechanism of baicalein on TiO_2_-MXene/PEDOT:PSS/GCE, the electrochemical kinetics were studied through measuring the CVs of 0.1 M of PB (pH 2.0) with 10.0 μM of baicalein at various scan rates in the range from 25 to 300 mV·s^−1^. As shown in Figure 7A, the redox currents increased significantly with the increase in scan rates, and the peak potentials shifted oppositely. Both the oxidation and reduction peak currents displayed good linearity against the scan rates. The relationship between the redox peak current and the scan rate is shown in the inset of Figure 7B with the corresponding linear regression equations of I_pa_ = 12.676 + 0.324 *v* (R^2^ = 0.991) and I_pc_ = 3.567 − 0.173 *v* (R^2^ = 0.997). These experimental results indicated that the redox reaction of baicalein on TiO_2_-MXene/PEDOT:PSS/GCE was an adsorption-controlled process.

**Scheme 1:**
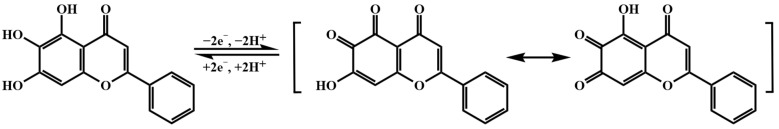
The proposed redox mechanism of baicalein on TiO_2_-MXene/PEDOT:PSS/GCE.

In addition, the peak potential (E_p_) versus the logarithm of scan rate (ln*v*) displayed a good linear curve (Figure 7C). The relevant linear regression equations were represented as E_pa_ = 0.331 + 0.029 ln*v* (R^2^ = 0.996) and E_pc_ = 0.499 − 0.021 ln*v* (R^2^ = 0.991). According to the Laviron formulas [30]:

(2) $$E_{\mathrm{pa}}=E^{0}+\frac{\mathrm{RT}}{\left(1-\alpha \right)\mathrm{nF}}\ln v$$

(3) $$E_{\mathrm{pc}}=E^{0}-\frac{\mathrm{RT}}{\alpha \mathrm{nF}}\ln v$$

where, E^0^ expresses the standard potential, α means the charge transfer coefficient and *n* represents electron transfer number. The values of α and *n* were calculated to be 0.58 and 2.1. The number of electrons in the baicalein redox reaction was equal to the number of protons [31]. The results proved that the redox reaction of baicalein on TiO_2_-MXene/PEDOT:PSS/GCE was a two-electron and two-proton redox process. The theoretical redox reaction mechanism is described in Scheme 1.

### 2.6. Electrochemical Detection of Baicalein by DPV

**Figure 8:**
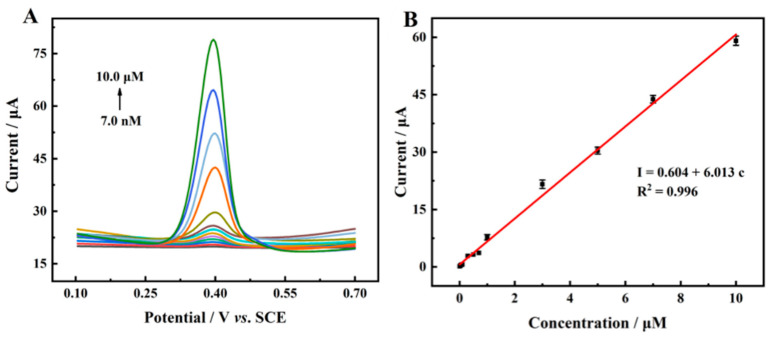
(**A**) DPVs of 0.007, 0.03, 0.05, 0.08, 0.1, 0.3, 0.5, 0.7, 1.0, 3.0, 5.0, 7.0 and 10.0 μM baicalein on TiO_2_-MXene/PEDOT:PSS/GCE in 0.1 M PB (pH 2.0); (**B**) Linear curve of oxidation peak current *vs.* concentration.

Under optimal experimental parameters, the detection performance of baicalein on TiO_2_-MXene/PEDOT:PSS/GCE was measured via DPV measurement. Figure 8A revealed the DPV response of baicalein in different concentrations in 0.1 M PB (pH 2.0). It was observed that as the baicalein concentration increased, the corresponding oxidation peak currents gradually strengthened. The baicalein concentration showed positive DPV linear dependence with its oxidation peak current response from 0.007 to 10.0 μM. The relevant linear equation (Figure 8B) between the DPV peak current response and the baicalein concentration was I (μA) = 0.604 + 6.013 c (μM) with R^2^ = 0.996. The LOD was calculated to be 2.33 nM (S/N = 3). To further explore the merits of TiO_2_-MXene/PEDOT:PSS/GCE, the detection limits and linear range of baicalein were compared by this system with those of previous electrochemical sensors. As shown in Table 1, the TiO_2_-MXene/PEDOT:PSS/GCE exhibited a lower LOD in contrast to the known related sensors. The excellent baicalein sensing performance of the TiO_2_-MXene/PEDOT:PSS/GCE was attributed to its high conductivity and superior adsorption ability originating from the synergies between materials.

The superior electrocatalysis performance in the detection of baicalein on TiO_2_-MXene/PEDOT:PSS/GCE was owing to the particular physicochemical properties of TiO_2_-MXene and PEDOT:PSS as well as the synergy effect of TiO_2_-MXene/PEDOT:PSS. In structural aspects, oxygen vacancy-rich TiO_2_ nanoparticles were uniformly distributed inside and on the surface of MXene. The heterostructure of TiO_2_-MXene provided electronic transport channels. Furthermore, the PEDOT:PSS film covered the surface of TiO_2_-MXene, which was beneficial for establishing connections between isolated individual TiO_2_-MXene nanosheets. The introduced PEDOT:PSS also improved the electrical conductivity of the composite, thus further supporting efficient electron transmission between the baicalein and electrode materials. Consequently, the TiO_2_-MXene/PEDOT:PSS composite was a new ideal electrode material for baicalein detection.

### 2.7. Repeatability, Reproducibility, Stability and Selectivity Studies 

**Figure 9:**
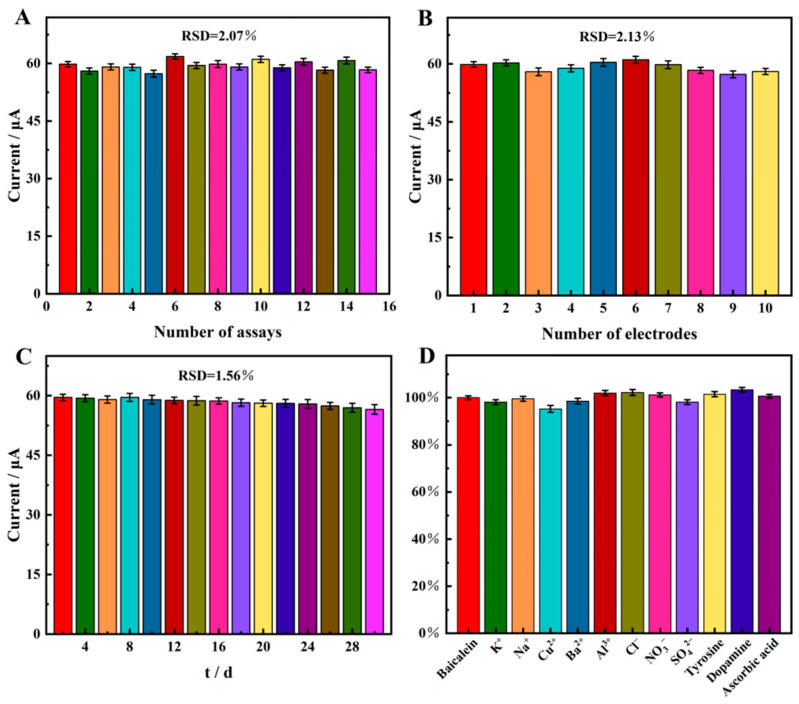
(**A**) Repetitive DPV responses of TiO_2_-MXene/PEDOT:PSS/GCE with the same electrode; (**B**) DPV responses of TiO_2_-MXene/PEDOT:PSS/GCE for ten parallel electrodes; (**C**) DPV responses of TiO_2_-MXene/PEDOT:PSS/GCE toward 10.0 μM baicalein in one month; (**D**) Selectivity studies of TiO_2_-MXene/PEDOT:PSS/GCE.

Repeatability, reproducibility, stability and selectivity were the basic parameters for investigating the practical capabilities of the fabricated electrochemical sensor. To assess the repeatability, one TiO_2_-MXene/PEDOT:PSS/GCE was utilized to detect 10.0 μM baicalein for fifteen repeated measurements (Figure 9A). The relative standard deviation (RSD) was 2.07%, which showed that TiO_2_-MXene/PEDOT:PSS/GCE had a favorable repeatability. To explore the reproducibility of electrochemical sensor, ten independent TiO_2_-MXene/PEDOT:PSS/GCEs were used to determine 10.0 μM baicalein, respectively. Figure 9B suggested that the RSD was calculated to be 2.13%, indicating that the proposed sensor had good reproducibility.

Furthermore, the stability of the prepared electrochemical electrode was investigated. The TiO_2_-MXene/PEDOT:PSS/GCE was assessed by storing the modified electrode at room temperature for one month and detecting 10.0 μM baicalein every two days. It was shown in Figure 9C that the RSD of the current response was only 1.56%, demonstrating the appreciable stability of TiO_2_-MXene/PEDOT:PSS/GCE.

Additionally, to verify the selectivity of the fabricated electrode, various potential interfering substances, including 50-fold of common ions (K^+^, Na^+^, Cu^2+^, Ba^2+^, Al^3+^, Cl^−^, NO_3_^−^, SO_4_^2−^) and 50-fold of tyrosine, dopamine and ascorbic acid, were evaluated in the presence of 10.0 μM baicalein. As shown in Figure 9D, the changes in the current response of interferences were below 5%. Although the concentrations of these interfering substances were much higher than that of baicalein (10.0 μM), there was no obvious influence on the peak current of baicalein detection. The result exhibited that the TiO_2_-MXene/PEDOT:PSS/GCE possessed a reliable selectivity. Based on the above electrochemical detection, a conclusion can be drawn that the TiO_2_-MXene/PEDOT:PSS/GCE demonstrated satisfactory repeatability, reproducibility, stability and selectivity in the detection of baicalein.

### 2.8. Real Sample Analysis

To verify the practicability of the developed electrochemical sensor in real samples, TiO_2_-MXene/PEDOT:PSS/GCE was used to determine baicalein in urine samples by the standard addition method via the DPV technique. The results of the analysis of baicalein are summarized in Table 2. The recoveries of baicalein were in the range of 98.0 to 102.0% (*n* = 5), and the RSDs value were all within 3.19%. All these results demonstrated that the TiO_2_-MXene/PEDOT:PSS composite modified electrode could be efficiently applied to practical samples.

## 3. Materials and Methods

### 3.1. Chemicals and Reagents

Ti_3_C_2_T_x_ MXene powder (56–59 wt%) was purchased from XFNANO Materials Tech Co., Ltd. (Nanjing, China). PEDOT:PSS (Clevios PH 1000, Net: 100 g = 100 mL) was obtained from Heraeus, Deutschland GmbH & Co. KG. (Leverkusen, Germany). NaH_2_PO_4_ (analytical grade, 99.0%) and Na_2_HPO_4_ (analytical grade, 99.0%) were acquired from Aladdin Reagent Co., Ltd. (Shanghai, China). H_3_PO_4_ (analytical grade, 85.0%) was purchased from Xilong Chemical Co., Ltd. (Shantou, China). Baicalein (analytical grade, 98.0%) was provided by Macklin Reagent Co., Ltd. (Shanghai, China). PB (0.1 M) was prepared with 0.1 M NaH_2_PO_4_, 0.1 M Na_2_HPO_4_ and H_3_PO_4_. All chemicals were used without any purification and the water used in this work was deionized water.

### 3.2. Instrumental Characterization

Scanning electron microscopy (SEM, Hitachi Company, Tokyo, Japan) and X-ray photoelectron spectroscopy (XPS, Escalab 250Xi, Thermo Fisher, Branchburg, NJ, USA) were used to understand the surface morphology and elemental composition of composite materials. All the electrochemical measurements were performed on a CHI760E electrochemical workstation (Shanghai, China). A standard three-electrode system was utilized for the electrochemical measurements, which consists of a saturated calomel electrode (SCE), a glass carbon electrode (GCE, 3 mm diameter) and a platinum wire electrode.

### 3.3. Synthesis of TiO_2_-MXene/PEDOT:PSS Composite

TiO_2_-MXene was prepared according to the procedure reported in the literature with slight modification [38]. Firstly, 0.2 g Ti_3_C_2_T_X_ was heated via a tube furnace to 200 °C with a heating rate of 3 °C·min^−1^ in an air atmosphere and maintained for 1 h. Then, the obtained sample was further calcined to 500 °C at a rate of 5 °C·min^−1^ in a tube furnace under flowing N_2_ and kept for 1 h.

TiO_2_-MXene/PEDOT:PSS composite was fabricated by a facile ultrasound method. That is, 1.0 mL of PEDOT:PSS and 10.0 mg of the prepared TiO_2_-MXene were dispersed into 9.0 mL deionized water. The deep blue suspension was ultrasound-sonicated for 1.0 h to obtain a dispersed solution.

### 3.4. Preparation of Modified Electrodes

**Scheme 2:**
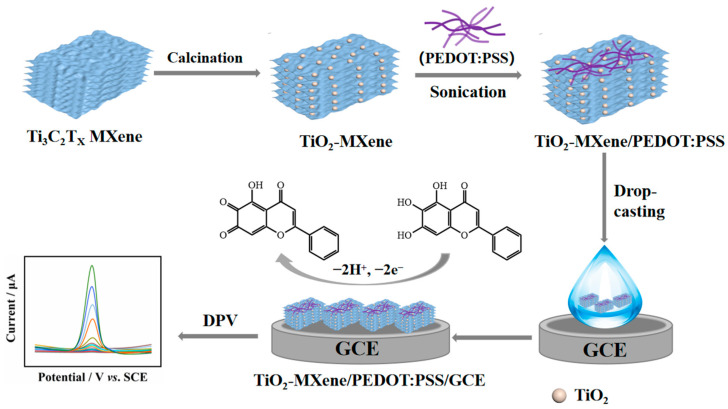
Schematic illustration for the preparation of TiO_2_-MXene/PEDOT:PSS/GCE and the sensing strategy for baicalein detection.

Prior to modification, the bare GCE was polished on the suede with 0.3 µm Al_2_O_3_ powder, then ultrasonically cleaned three times in deionized water and ethanol, respectively, for 1 min. Subsequently, 5.0 μL of the above TiO_2_-MXene/PEDOT:PSS dispersion was drop-coated onto bare GCE and then dried under an infrared lamp to obtain TiO_2_-MXene/PEDOT:PSS/GCE. TiO_2_-MXene/GCE was manufactured in the same step (1 mg/mL TiO_2_-MXene). The synthetic process of TiO_2_-MXene/PEDOT:PSS/GCE and its sensing mechanism for the detection of baicalein were presented in Scheme 2.

### 3.5. Electrochemical Measurement

Electrochemical impendence spectroscopy (EIS) measurements were investigated in 5.0 mM [Fe(CN)_6_]^3−/4−^ containing 0.1 M KCl with a frequency from 1 to 10^5^ Hz, and the amplitude was 5 mV. Cyclic voltammetry (CV) and differential pulse voltammetry (DPV) measurements were used to investigate the electrochemical behaviors of a series of electrodes in 0.1 M PB, with a potential range of 0.1 to 0.7 V and at a scan rate of 100 mV·s^−1^.

### 3.6. Preparation of Real Samples

The urine samples were collected from healthy volunteers. The obtained urine sample was filtered by a 0.45 μm pore-size nylon filter and diluted with 0.1 M PB (pH = 2.0) 100 times. Baicalein samples with known standard concentrations were used to spike the urine samples for the real sample analysis using the standard addition method.

## 4. Conclusions

In summary, our work reported a novel electrochemical sensing platform for baicalein determination, which was developed on the grounds of the synergistic mechanism between TiO_2_-MXene and PEDOT:PSS. The TiO_2_-MXene/PEDOT:PSS composite presented high conductivity, a large electroactive surface area, and superior electrocatalytic activity, which were effective for electron transfer and the adsorption of baicalein. Based on these characteristics of the composite material, the established electrochemical sensor also exhibited a wide linear response of 0.007–10.0 μM and a low LOD of 2.33 nM. At the same time, the as-prepared electrode demonstrated excellent repeatability, reproducibility and stability for the detection of baicalein. Additionally, this sensor further demonstrated its satisfactory detection ability in human urine samples, which presented good spiked recoveries, indicating great potential application prospects in the electrochemical detection of baicalein. 

## Figures and Tables

**Table 1:** Analytical comparison of other previously reported electrochemical sensors for baicalein determination.

Modified Electrodes	Method	Linear Range (μM)	Detection Limit (nM)	Reference
Ta_2_O_5_-Nb_2_O_5_@CTS ^a^/CPE ^b^	DPV	0.08–8	50	[32]
TRGO ^c^/GCE	DPV	0.01–1; 1–10	6	[33]
Au-Pt@BPC ^d^/CILE ^e^	DPV	0.48–2; 4–140	10	[34]
D-Ti_3_C_2_T_x_/C_60_ ^f^/GCE	DPV	0.015–4.0	5	[35]
BDD ^g^ electrode	SWV ^h^	1–95	260	[36]
Ta_2_O_5_-CTS ^i^/CPE	LSV ^j^	0.08–4	50	[37]
TiO_2_-MXene/PEDOT:PSS/GCE	DPV	0.007–10.00	2.33	This work

Note: ^a^: tantalum oxide, niobium oxide and chitosan; ^b^: carbon paste electrode; ^c^: thermally reduced graphene oxide; ^d^: gold-platinum bimetal decorated biomass porous carbon; ^e^: carbon ionic liquid electrode; ^f^: delaminated titanium carbide nanosheet and fullerene; ^g^: boron doped diamond; ^h^: square wave voltammetry; ^i^: tantalum oxide and chitosan; ^j^: linear scan voltammetry.

**Table 2:** The recoveries of the TiO_2_-MXene/PEDOT:PSS/GCE in urine samples (*n* = 5).

Sample	Added (μM)	Found (μM)	Recovery (%)	RSD (%)
1	0	-	-	-
2	0.5	0.49 ± 0.02	98.0	3.19
3	1	1.02 ± 0.03	102.0	2.31
4	3	3.05 ± 0.09	101.7	2.18
5	5	4.93 ± 0.13	98.6	1.79

## Data Availability

The data presented in this study are available in the article.

## Sample Availability

Compound samples are not available from the authors.
